# Effects of physician‐present prehospital care in patients with out‐of‐hospital cardiac arrest on return of spontaneous circulation: A retrospective, observational study in Saga, Japan

**DOI:** 10.1002/hsr2.1981

**Published:** 2024-04-22

**Authors:** Kota Shinada, Ayaka Matsuoka, Toru Miike, Hiroyuki Koami, Yuichiro Sakamoto

**Affiliations:** ^1^ Department of Emergency and Critical Care Medicine, Faculty of Medicine Saga University Saga City Japan

**Keywords:** advanced airway management, emergency medical service, epinephrine, on‐site operation time

## Abstract

**Background and Aims:**

Emergency medical services for out‐of‐hospital cardiac arrest (OHCA) vary according to region and country, and patient prognosis differs accordingly. In Japan, physicians may provide prehospital care. However, the effect of physician‐present prehospital care on achieving return of spontaneous circulation (ROSC) in patients with cardiac arrest is not clear. Here, we aimed to examine the effect of physician‐present prehospital care on the prognosis of patients with OHCA at our hospital compared with physician‐absent care.

**Methods:**

In this retrospective, observational study, patients aged ≥18 years with non‐traumatic OHCA from a single center in Saga City, Japan, between April 2011 and December 2019, were included. Patients were divided into two groups, based on prehospital physician presence or absence. Logistic regression analysis was used to determine the association between physician‐present prehospital care and ROSC.

**Results:**

Of 820 patients with OHCA, 151 had a physician present and 669 did not. Logistic regression analysis with no adjustment showed that the odds ratio (OR) of physician‐present prehospital care for an increased ROSC rate was 1.74 (95% confidence interval [CI]: 1.22−2.48, *p* = 0.002). Logistic‐regression analysis adjusted for ROSC‐related factors indicated an OR of 1.05 (95% CI: 0.47−2.34, *p* = 0.914) for physician‐present prehospital care to ROSC.

**Conclusion:**

Physician‐present prehospital care may not necessarily lead to increased ROSC rates. However, insufficient data limited our study findings. Further studies involving larger sample sizes are warranted.

## Key points

- We examined the effects of physician‐present prehospital care on the prognosis of patients with out‐of‐hospital cardiac arrest (OHCA) compared with physician‐absent care.
- Physician‐present prehospital care was not necessarily helpful in achieving the return of spontaneous circulation (ROSC) when adjusting for all influencing factors.
- The 95% confidence interval of physician‐present prehospital care to ROSC straddled 1 when adjusted for factors such as prehospital advanced airway usage, prehospital epinephrine administration, and on‐site operation time.

## 1 INTRODUCTION

Emergency medical service (EMS) systems for critically ill patients, including patients with out‐of‐hospital cardiac arrest (OHCA), vary from region to region and from country to country.^1^ These differences lead to differing prognoses for patients with OHCA.^2^, ^3^

Various studies have examined prehospital patient care in terms of prognosis and outcomes for patients with OHCA, with several studies having reported the effects of prehospital epinephrine administration and advanced airway management as components of prehospital care in OHCA; however, conflicting evidence has been reported concerning the advantages and disadvantages of these measures.^4^, ^5^, ^6^, ^7^, ^8^, ^9^, ^10^, ^11^, ^12^, ^13^, ^14^, ^15^, ^16^ In patients with OHCA, prehospital epinephrine administration and advanced airway management may prolong cardiopulmonary resuscitation (CPR) time and negatively impact prognosis due to delayed hospital arrival.^17^ Other studies have examined the effect of physician‐present prehospital care on the prognosis of OHCA. These studies reported that prehospital physician‐present care was associated with improved prehospital outcomes, survival to discharge, and neurologic prognosis.^18^, ^19^, ^20^, ^21^, ^22^ However, such studies are few. In addition, these studies have differing protocols for physician‐present and physician‐absent (EMS only) prehospital care. Because different regions have different EMS systems, the effectiveness of physician‐present prehospital care can be expected to vary according to region, and the results of these studies are not applicable to all countries or regions.

In Japan, prehospital care involves dispatching physicians and nurses to the scene to provide prehospital care in combination with an emergency medical team.^23^

In Japan, EMS personnel can only provide limited on‐site care for OHCA. In the Saga region of Japan, EMS personnel are not allowed to administer epinephrine to a patient with an unwitnessed OHCA and those in asystole. When physician‐present prehospital care is provided, all patients with OHCA are candidates for advanced airway usage and epinephrine administration. In Saga Prefecture, where prehospital care for OHCA is provided under such protocols, examining the utility of physician‐present prehospital care may lead to improvements in prehospital activity protocols. Therefore, this study aimed to examine the effect of physician‐present prehospital care on the prognosis of patients with OHCA at our hospital compared with physician‐absent care.

## 2 METHODS

### 2.1 Study design and participants

This single‐center, retrospective, observational study was conducted at Saga University Hospital. Inclusion criteria were patients aged ≥18 years with non‐traumatic OHCA who had been transported to our hospital between April 2011 and December 2019. Exclusion criteria were patients in cardiac arrest after EMS contact, those with a confirmed intention not to resuscitate during transport or upon arrival, those transported by helicopter, and those with missing data. Patient data were extracted from their medical records for analysis.

This study was approved by the Ethics Committee of Saga University Hospital (approval no.: 2023‐02‐R‐07) and conforms to the provisions of the Declaration of Helsinki, as revised in Fortaleza, Brazil, in October 2013. The need for patient informed consent for participation and publication was waived by the Ethics Committee of Saga University Hospital owing to the study's retrospective design.

### 2.2 Demographic information concerning the Saga area

There are five fire departments in Saga Prefecture; this hospital is located within the jurisdiction of one of them, the Saga Regional Fire Department, which covers an area of approximately 790 km^2^ including 350,000 citizens. Emergency dispatch services deal with 15,000 cases per year.

### 2.3 Physician‐absent (standard EMS) prehospital care

In the absence of a physician, that is, standard EMS, EMS personnel are contacted from the communications command center and dispatched from the nearest fire station. In circumstances of standard EMS, certified EMS personnel can perform peripheral venous cannulation and epinephrine administration under the direction of a physician via telephone, including for patients with OHCA, excluding those with an asystole rhythm and no witnesses. EMS personnel are permitted to attempt an intravenous route twice per patient. In addition, an advanced airway technique can be used under the direction of a physician via telephone.

### 2.4 Physician‐present prehospital care

Physicians are also dispatched to the scene for patients in serious condition, including patients whose cardiac arrest occurs within the relevant medical area. The physician team mostly comprises a driver, a navigator, a physician, and a nurse, and operates on week days from 09:00 to 17:00 h. The physician and nurse routinely respond to critical cases, including those of cardiac arrest, as part of their work in the hospital. The driver and navigator are assigned from the fire department and work as part of the standard EMS team daily. The driver and navigator function both as physician team personnel and as communicators with the field EMS, ensuring field activities run smoothly. The need for physician dispatch is determined by the communication command center. If the content of the emergency call is suggestive of a cardiac arrest, a dispatcher may request the dispatch of a physician to the scene at the same time as the request for the dispatch of a standard EMS team. Physicians are on standby at the emergency medical center and will attend a scene with resuscitation equipment when requested. In such situations, prehospital epinephrine administration, advanced airway usage, and determining the cause of an OHCA can commence at the discretion of the on‐site physician. Unlike the EMS alone circumstance, there are no restrictions on the use of prehospital epinephrine administration or the number of attempts needed to obtain an intravenous route.

### 2.5 Data collection

We collected data concerning patient characteristics such as sex, age, witness, bystander CPR, and defibrillation with an automated external defibrillator, initial rhythm (shockable rhythm or not), prehospital care (epinephrine administration, advanced airway usage, and physician involvement), and outcomes (return of spontaneous circulation [ROSC], 1‐month survival, and 1‐month survival with a Glasgow−Pittsburgh cerebral performance category [CPC] 1–2). On‐site operation time was also measured. We defined on‐site operation time as the time from physician or EMS contact with a patient to departure from the scene. We included interrupted transport and intervention times in the on‐site operation time.

### 2.6 Statistical analysis

Patients were divided into two groups according to the prehospital presence or absence of a physician. Univariate analysis was performed to compare patient characteristics. Categorical variables were compared using a Fisher's exact test. The mean and median differences for continuous variables between the two groups were evaluated using independent Student's *t*‐ and Wilcoxon rank sum tests. The normality of the distribution for continuous variables was assessed using a Shapiro–Wilk test. Normally distributed data are expressed as means (standard deviations), whereas non‐normally distributed data are expressed as medians (interquartile ranges).

Patients were further divided into two groups according to the presence or absence of ROSC and patient characteristics were then compared in a similar manner.

Logistic regression analysis was performed to analyze the effect of physician‐present prehospital care on ROSC in patients with OHCA; Model 1 shows the odds ratio (OR) of physician‐present prehospital care on ROSC in OHCA with no adjustment. In Model 2, age, sex, bystander witness, bystander CPR, bystander defibrillation, presence of shockable rhythm, cardiac origin, time of occurrence, and location of occurrence were used as adjustment factors. In addition, Model 3 used prehospital advanced airway usage, prehospital epinephrine administration, and on‐site operation time as adjustment factors in addition to those used in Model 2.

Furthermore, to examine the effect of physician‐present EMS on ROSC, stratified analysis was performed in the subgroups in terms of whether a patient in cardiac arrest was witnessed by a bystander and whether the electrocardiogram waveform was shockable at the time of EMS contact.

All statistical analyses were performed using JMP Pro Version 14 package software (SAS Inc.). The significance threshold was set at *p* < 0.05 (two‐sided).

## 3 RESULTS

**Figure 1:**
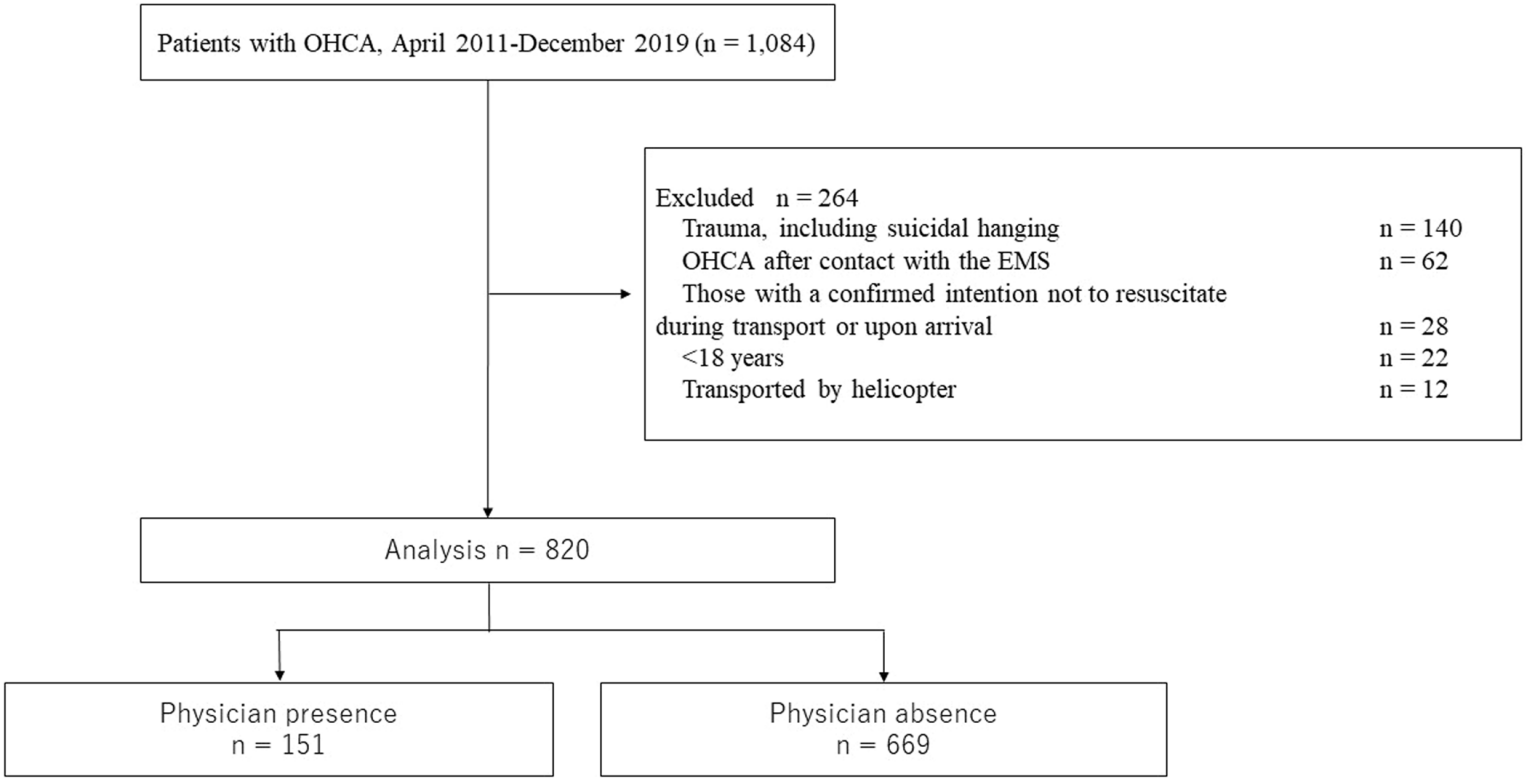
Patients with OHCA during the study period and patients included in the analysis. CPR, cardiopulmonary resuscitation; EMS, emergency medical services; OHCA, out‐of‐hospital cardiac arrest.

During the approximate 9‐year study period, resuscitation was attempted in 1084 patients with OHCA. We excluded trauma patients, those in cardiac arrest after EMS contact, those with a confirmed intention not to resuscitate during transport or upon arrival, those aged <18 years, and those who had been transported to the hospital by helicopter. Finally, 820 patients were included in the analysis. Of these, physicians were present for 151 patients and absent for 669 patients (Figure 1).

Patient characteristics of groups divided according to the prehospital presence or absence of a physician are shown in Table 1. There were no significant differences in the ratios in relation to age, sex, bystander witness, bystander CPR, bystander defibrillation, and shockable rhythm. The ratios for prehospital advanced airway usage and prehospital epinephrine administration were higher in the physician‐present group (prehospital advanced airway usage: 140 [92.7%] vs. 71 [10.6%]; *p* < 0.001, prehospital epinephrine administration: 141 [93.4%] vs. 32 [4.8%]; *p* < 0.001). Regarding the prehospital advanced airway approach, there were 137 tracheal intubations, 1 supraglottic airway device, and 2 cricothyrotomies in the physician‐present group. The physician‐absent group had 1 tracheal intubation and 70 supraglottic airway devices. The success rate of establishing venous access in the physician‐absent group was 33/67 (49.3%). There was no significant difference in the ratio in terms of cardiac origin. Regarding time of arrest, the ratio for daytime OHCA was higher in the physician‐present group (151 [100.0%] vs. 472 [70.6%]; *p* < 0.001). Regarding location of arrest, the ratio of home was lower and the ratios of public area and other locations were higher in the physician‐present group (home: 91 [60.3%] vs. 495 [74.0%]; *p* = 0.001, public area: 47 [31.1%] vs. 152 [22.7%]; *p* = 0.035, other: 9 [6.0%] vs. 12 [1.8%]; *p* = 0.008). There were no significant between‐group differences in ratios concerning cardiac arrests at work. On‐site operation time was longer in the physician‐present group (14 [11–18] vs. 10 [8–13]; *p* < 0.001). The ratio of ROSC was higher in the physician‐present group (83 [55.0%] vs. 276 [41.3%]; *p* = 0.003), but there were no significant differences in 1‐month survival and 1‐month survival with CPC 1–2.

**Table 1:** Characteristics of patients with OHCA according to physician presence.

		Total (*n* = 820)	Physician‐present (*n* = 151)	Physician‐absent (*n* = 669)	*p* Value
Age		78 (66–86)	79 (67–87)	78 (66–86)	0.518
Female		335 (40.9%)	65 (43.1%)	270 (40.4%)	0.583
Bystander‐witnessed		286 (34.9%)	63 (41.7%)	223 (33.3%)	0.059
Bystander CPR		400 (48.8%)	71 (47.0%)	329 (49.2%)	0.653
Bystander defibrillation		5 (0.6%)	2 (1.3%)	3 (0.5%)	0.230
Shockable rhythm		82 (10.0%)	20 (13.3%)	62 (9.3%)	0.175
Prehospital advanced airway usage		211 (25.7%)	140 (92.7%)	71 (10.6%)	<0.001
Prehospital epinephrine administration		173 (21.1%)	141 (93.4%)	32 (4.8%)	<0.001
Cardiac origin		226 (27.6%)	43 (28.5%)	183 (27.4%)	0.763
Time of arrest					
	Daytime (7:00–22:59 h)	623 (76.0%)	151 (100.0%)	472 (70.6%)	<0.001
	Night‐time (23:00–6:59 h)	197 (24.0%)	0 (0.0%)	197 (29.5%)	<0.001
Location of arrest					
	Home	586 (71.5%)	91 (60.3%)	495 (74.0%)	0.001
	Work	14 (1.7%)	4 (2.7%)	10 (1.5%)	0.304
	Public area	199 (24.3%)	47 (31.1%)	152 (22.7%)	0.035
	Other	21 (2.6%)	9 (6.0%)	12 (1.8%)	0.008
On‐site operation time (min)		11 (9−13)	14 (111−8)	10 (81−3)	<0.001
ROSC		359 (43.8%)	83 (55.0%)	276 (41.3%)	0.003
One‐month survival		46 (5.6%)	10 (6.6%)	36 (5.4%)	0.557
One‐month survival with CPC 1–2		25 (3.1%)	2 (1.3%)	23 (3.4%)	0.290

Abbreviations: CPC, cerebral performance category; CPR, cardiopulmonary resuscitation; ROSC, return of spontaneous circulation.

Differences in patient characteristics between groups, when divided according to ROSC (ROSC and non‐ROSC groups), are shown in Supporting Information S1: Table 1. The ratios of bystander witness and shockable rhythm were higher in the ROSC group (bystander‐witnessed: 186 [51.8%] vs. 100 [21.7%]; *p* < 0.001, shockable rhythm: 68 [18.9%] vs. 14 [3.0%]; *p* < 0.001). There were no significant differences in terms of age, sex, bystander CPR, and bystander defibrillation. The ratios in terms of physician presence, prehospital advanced airway usage, and prehospital epinephrine administration were higher in the ROSC group (physician presence: 83 [23.1%] vs. 68 [14.8%]; *p* = 0.003, prehospital advanced airway usage: 106 [29.5%] vs. 105 [22.8%]; *p* = 0.030, prehospital epinephrine administration: 98 [27.3%] vs. 75 [16.3%]; *p* < 0.001). The ratio in terms of cardiac origin was lower in the ROSC group (243 [67.7%] vs. 351 [76.1%]; *p* = 0.008). Regarding the time of arrest, the ratio in terms of daytime OHCA was higher in the ROSC group (285 [79.4%] vs. 338 [73.3%]; *p* = 0.048).

Logistic‐regression analysis was used to analyze the effect of physician presence on ROSC (Table 2). The unadjusted OR was 1.74 (1.22−2.48, *p* = 0.002). In Model 2, the OR was 1.52 (1.02−2.28, *p* = 0.040) when adjusted for the factors specified regardless of physician presence (age, female, bystander‐witnessed, bystander CPR, bystander defibrillation, shockable rhythm, cardiac origin, time of arrest, and home). In Model 3, the OR was 1.05 (0.47−2.34, *p* = 0.914) when adjusted for Model 2 and the factors specified according to physician presence (prehospital advanced airway usage, prehospital epinephrine administration, and on‐site operation time).

**Table 2:** ROSC improvement effect of physician presence.

		OR	95% CI	*p* Value
	Physician‐absent	Physician‐present		
Model 1 (crude)	ref = 1	1.74	1.22−2.48	0.002
Model 2	ref = 1	1.52	1.02−2.28	0.040
Model 3	ref = 1	1.05	0.47−2.34	0.914

Abbreviations: CI, confidence interval; CPR, cardiopulmonary resuscitation; OR, odds ratio; ROSC, return of spontaneous circulation.

Model 1: crude values are unadjusted.

Model 2: adjusted for age, female, bystander‐witnessed, bystander CPR, bystander defibrillation, shockable rhythm, cardiac origin, time of arrest, and home.

Model 3: adjusted for the factors of Model 2 and prehospital advanced airway usage, prehospital epinephrine administration, and on‐site operation time.

Table 3 shows the results of subgroup analyses conducted to analyze the effect of physician presence on ROSC. In the group showing shockable ECG waveforms, the OR was 5.04 (0.62−41.24, *p* = 0.131) with no adjustment and 1.60 (1.09−2.34, *p* = 0.016) when non‐shockable. For Model 2, the ORs were 9.23 (0.75−113.89, *p* = 0.083) and 1.45 (0.96−2.2, *p* = 0.080), respectively. For Model 3, the ORs were 1.58 (0.01−404.48, *p* = 0.872) and 0.89 (0.38−2.11, *p* = 0.791), respectively. For the bystander‐witnessed group, the OR was 1.6 (0.86−2.98, *p* = 0.135) with no adjustment, and 1.65 (1.03−2.64, *p* = 0.036) for non‐bystander‐witnessed group. For Model 2, the ORs were 1.66 (0.85−3.25, *p* = 0.141) and (0.89−2.42, *p* = 0.136), respectively. For Model 3, the ORs were 1.54 (0.49−4.83, *p* = 0.463) and 0.64 (0.182−2.25, *p* = 0.490), respectively.

**Table 3:** Logistic‐regression analysis for ROSC in subgroups.

		Physician‐present					
		OR (95% CI)		*p* value	OR (95% CI)		*p* value
	Physician‐absent	Shockable (*n* = 82)			Non‐shockable (*n* = 738)		
Model 1 (crude)	ref = 1	5.04	0.62−41.24	0.131	1.60	1.09−2.34	0.016
Model 2	ref = 1	9.23	0.75−113.89	0.083	1.45	0.96−2.20	0.080
Model 3	ref = 1	1.58	0.014−04.48	0.872	0.89	0.38−2.11	0.791

		Witnessed (*n* = 286)			Unwitnessed (*n* = 534)		
Model 1 (crude)	ref = 1	1.6	0.86−2.98	0.135	1.65	1.03−2.64	0.036
Model 2	ref = 1	1.66	0.85−3.25	0.141	1.47	0.89−2.42	0.136
Model 3	ref = 1	1.54	0.49−4.83	0.463	0.64	0.18−2.25	0.490

Abbreviations: CI, confidence interval; CPR, cardiopulmonary resuscitation; OR, odds ratio; ROSC, return of spontaneous circulation.

Model 1: crude values are unadjusted.

Model 2: adjusted for age, female, bystander‐witnessed, bystander CPR, bystander defibrillation, shockable rhythm, cardiac origin, time of arrest, and home.

Model 3: adjusted for the factors of Model 2 and prehospital advanced airway usage, prehospital epinephrine administration, and on‐site operation time.

## 4 DISCUSSION

Logistic‐regression analysis showed that physician‐present prehospital care was not necessarily helpful in achieving ROSC when adjusting for all factors that might have influenced ROSC and that the 95% CI of physician‐present prehospital care to ROSC straddled 1 when adjusted for factors such as prehospital advanced airway usage, prehospital epinephrine administration, and on‐site operation time. In the present study, prehospital advanced airway usage and prehospital epinephrine administration were performed simultaneously in many cases, making it difficult to determine the effect of each on ROSC. Prehospital advanced airway usage and prehospital epinephrine administration as statistical correction factors may also have contributed to statistical instability since most cases in the physician‐present group involved both interventions. Whether prehospital epinephrine administration and advanced airway usage improve the prognosis of patients with OHCA remains controversial,^4^, ^5^, ^6^, ^7^, ^8^, ^9^, ^10^, ^11^, ^12^, ^13^, ^14^, ^15^, ^16^ and further data are needed for more detailed studies. The success rate of EMS in establishing venous access in this study was 49.3%. In a previous study, the success rate in another region of Japan was reported to be 63.8%,^24^ and compared to this report, the success rate in our region is slightly lower. This might also have affected the results.

In patients with OHCA, ROSC is the primary goal of initial CPR.^25^ However, the significant human and economic costs of intensive care after CPR suggest that patients with good neurologic prognosis should be identified early, and priority should be given to intensive care of such patients.^26^ In this single‐center, retrospective, validation study, it was not possible to assess long‐term prognosis, such as survival at discharge, due to insufficient data. Further studies with larger sample sizes are needed to analyze the effect of physician‐present prehospital care on neurological outcomes in patients with OHCA. Prehospital activities for OHCA treatment vary around the world.^1^ In some areas, resuscitation is continued prehospital and patients are transported to the hospital only after ROSC has been achieved; therefore, the decision to stop resuscitation for cases in which ROSC cannot be achieved is made in the prehospital setting. This type of activity may lead to the optimization of medical resources. During the study period, physicians did not terminate resuscitation at the scene for non‐traumatic OHCA patients unless all six criteria suggestive of apparent death were present: no response to pain stimulus, no respiration, no palpable carotid artery, dilated pupils and no light reflex, cold sensation, and rigor mortis or postmortem lividity. These six criteria are consistent with those used by EMS to determine patients not to be transported to the hospital. Besides early medical intervention aimed at saving lives, deresuscitation and the selection of hospitals to transport patients to may also be important aspects of the role of physicians dispatched to the scene.

In this study, there was a significant difference in the location of OHCA between the physician‐present and physician‐absent groups. The fact that the physician team operates only during daytime on week days may be the reason for the high number of trips to work and public places. Additionally, during daytime, patients are often transported to nearby hospitals, but during nighttime, this hospital often accepts them.

We consider that our findings concerning physician‐present prehospital care prolonging on‐site operation time are important. Prehospital epinephrine administration delays hospital arrival and may affect the prognosis of patients with OHCA.^17^ In the present study, the on‐site operation time was significantly prolonged in the physician‐present group. The physician team includes members who work as part of the standard EMS team on a daily basis. They facilitate the on‐site activities of the physician team by working with EMS in the field. Therefore, the prolonged time spent at the scene in the physician‐present group may be caused by prehospital advanced airway usage and prehospital epinephrine administration. The association between longer on‐site operation time and ROSC was not determined in this study; however, assessing such a possible association may provide important information for improving prehospital care protocols for OHCA.

When we compared between ROSC and non‐ROSC groups, significant differences were observed in shockable rhythm and bystander‐witness percentages. There was no significant difference in the percentage of shockable waveforms or bystander‐witnessing between the physician‐present group and the physician‐absent group. However, the shockable and bystander‐witnessed groups may have been more likely to achieve ROSC: thus, we performed a stratified analysis. In the stratified analysis, when adjusting for factors that may contribute to ROSC, the OR of physician presence in relation to ROSC was not statistically significant in both the shockable and bystander‐witnessed groups, indicating that physician presence might not be associated with ROSC in these subgroups. In other words, physician presence did not increase ROSC rates in these subgroups. However, the data in relation to this subgroup analysis was limited, with concerns regarding statistical instability. Therefore, it remains unclear based on our study findings whether this specific analysis showed clinically valid results.

Our study had some limitations. First, some of the patients who obtained ROSC were found to have an initially good neurologic prognosis in this single‐center study; however, it was not possible to evaluate these patient populations in the long‐term. Second, the experience and related skills of the physicians and paramedics may have differed, such that the effect of physician presence on increasing ROSC rates could have been influenced by their varying experience and skills. Third, there were insufficient data to perform subgroup analyses.

## 5 CONCLUSIONS

In summary, physician‐present prehospital care may not necessarily lead to increased ROSC rates. However, the limitation of this study was that there was insufficient data to identify the subgroups in which physician presence was beneficial, and further studies are warranted.

## AUTHOR CONTRIBUTIONS

**Kota Shinada**: Conceptualization; data curation; formal analysis; investigation; methodology; project administration; writing—original draft; writing—review and editing. **Ayaka Matsuoka**: Conceptualization; data curation; formal analysis; investigation; methodology; writing—original draft; writing—review and editing. **Toru Miike**: Conceptualization; data curation; formal analysis; investigation; methodology; writing—review and editing. **Hiroyuki Koami**: Conceptualization; writing—review and editing. **Yuichiro Sakamoto**: Conceptualization; supervision; writing—review and editing.

## CONFLICT OF INTEREST STATEMENT

The authors declare no conflict of interest.

## TRANSPARENCY STATEMENT

The lead author Kota Shinada affirms that this manuscript is an honest, accurate, and transparent account of the study being reported; that no important aspects of the study have been omitted; and that any discrepancies from the study as planned (and, if relevant, registered) have been explained.

## Supporting information

Supporting information.

## Data Availability

The data that support the findings of this study are available from the corresponding author upon reasonable request.
